# On The Structure‐Property‐Relationships of Textile Coated and Mechanical Recycled Polycarbonate Based Thermoplastic Blends for Automotive Interior Applications

**DOI:** 10.1002/marc.202500165

**Published:** 2025-04-30

**Authors:** Christoph Callsen, Julian Hampl, Bernd Trinkwalter, Thomas Meins

**Affiliations:** ^1^ Motherson DRSC Deutschland GmbH Germany; ^2^ Institute for Circular Economy of Bio:Polymers at Hof University (ibp) Hof University of Applied Sciences Germany

**Keywords:** automotive interior parts, mechanical recycling, PC/PET blend, single‐screw compounding, structure‐property relationship

## Abstract

This work introduces a convenient low technology recycling process of textile covered bezels based on polycarbonate thermoplastic materials. The recycled polymers are suitable starting materials for the reuse in automotive interior applications. Specifically, this work focuses on changes in thermal, morphological, rheological and mechanical properties caused by the recycling process. Fabric back‐injected polycarbonate (PC) based blends are semi‐automatically produced, shredded, pre‐dried and granulated using a single‐screw extruder. TEM images of the recycled materials show a fine droplet phase distribution of textile polyethylene terephthalate (PET) in PC, PC blend respectively, as well as the formation of a PET matrix in a neat PC/PET blend. DSC analyses indicated a shift in glass transition temperatures, suggesting partial miscibility of the blends. Compounding of textile PET with PC based blends led to decrease in viscosity at 280 °C. The blends show a slight decrease in strength and strain at break, due to hindered plastic deformation in PC blends, but increase of strength and strain at break of neat PC/PET due to PET becoming the matrix phase. This work highlights the potential of a partial miscibility of textile PET and polycarbonate which is used for a novel recycling approach for textile covered bezels contributing to sustainability by reducing waste and costs.

## Graphical abstract

An advanced low‐technology recycling process converts textile covered bezels into reusable materials for automotive interiors. The back‐injected bezels are shredded and regranulated. Thermal, morphological, rheological, and mechanical property changes are analyzed. Blending PC‐based polymers with the PET‐fabric results in nanoscale droplet morphologies in the Matrix. The mechanical properties remain within an acceptable range. This work promotes sustainability through waste reduction.

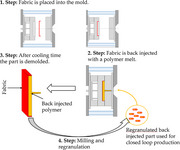

## 1 Introduction

**Figure 1:**
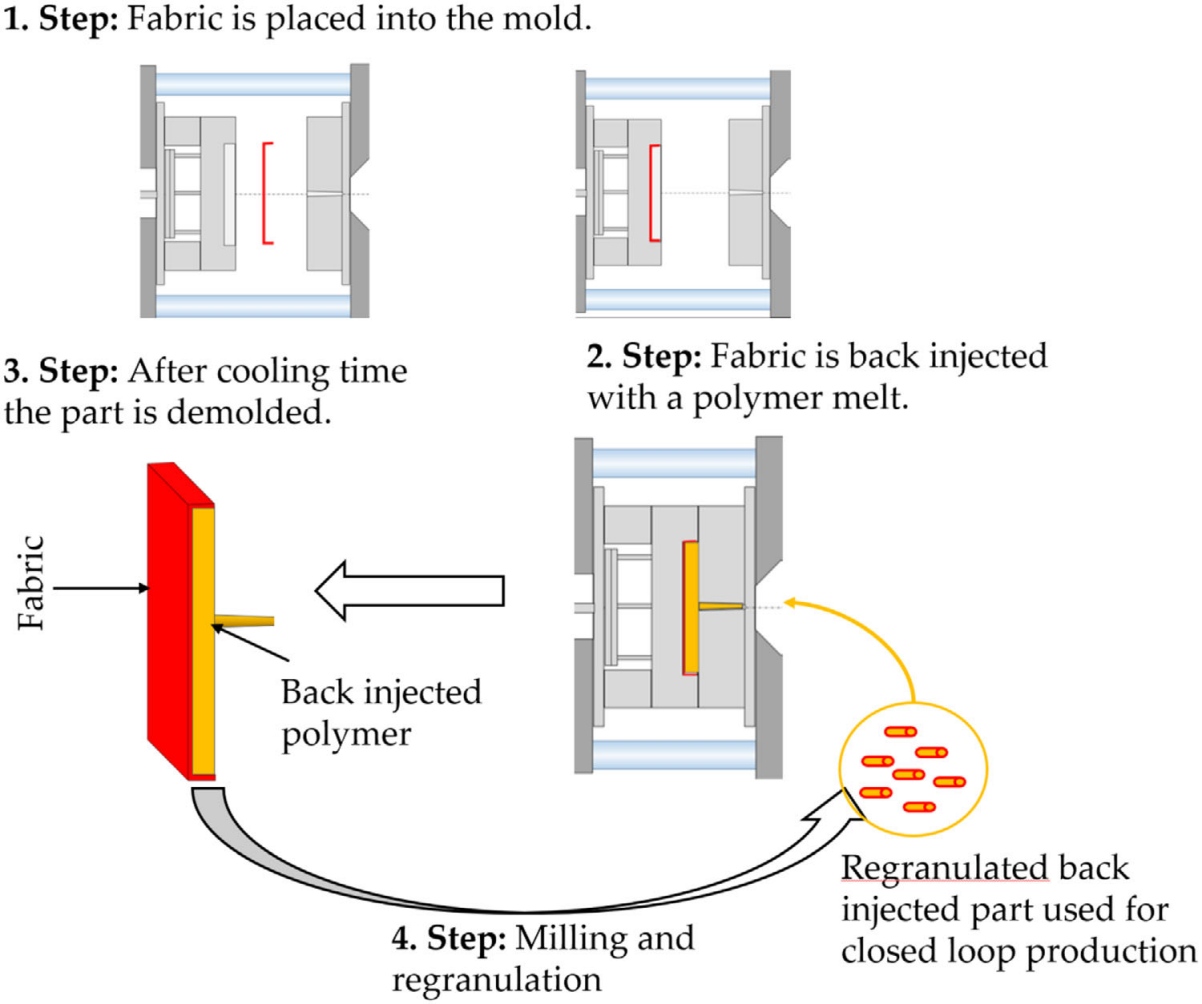
Schematic representation of the back‐injection process used for the production of textile covered bezels. Step 4 illustrates the recycling step investigated in this work in order to create a circular production.

The European Commission's press release of June 13, 2023 on improving the design and end‐of‐life management of motor vehicles, the so‐called ELV Directive, aims to ensure that 25% post consumer recyclate (PCR) must be used in newly built vehicles from 2030.^[^ ^1^ ^]^ Approximately 150–200 kg of plastic is used in current vehicles, which corresponds to ≈12%–15% of the vehicle weight.^[^ ^2^ ^]^ The proportion of plastics in cars will continue to grow due to the increasing demand for ever lighter, more fuel‐efficient, and at the same time more powerful vehicles.^[^ ^3^ ^]^ Furthermore, both the number of electric vehicles and the level of autonomous driving are rising sharply. The further development of autonomous driving means that the interior of the vehicle is increasingly becoming a third living space. In the future, car interiors will therefore have to offer alternative seating positions for working and relaxing, resulting in more and more functional and decorative surfaces.^[^ ^4^, ^5^ ^]^ At the same time, due to various sustainability strategies and a general increase in environmental awareness, the proportion of recycled plastics, so‐called recyclates, is being greatly increased in development and design and in some cases specified by various OEMs (Original Equipment Manufacturers). Values of 20%–25% of recycled materials are expected by 2025 in some cases.^[^ ^6^, ^7^ ^]^ In addition to commonly recycled plastics such as Polypropylene (PP) or Polyethylene (PE), there are other materials and material types used in vehicle interiors. This includes engineering plastics such as polycarbonate (PC), acrylonitrile butadiene styrene (ABS), polyethylene terephthalate (PET) and their corresponding blends. These types have both the necessary thermal resistance and a very high impact strength, which are essential for use in the head and knee impact area. Furthermore, the proportion of technical and decorative textiles in the interior is set to increase to 35 kg per vehicle.^[^ ^8^ ^]^ These must be taken into account in recycling quotas, requirements and implementation regarding sustainability issues. Polyester, especially PET, is one of the most commonly used artificial fiber types.^[^ ^9^ ^]^ In order to use these textiles for interior trim applications, the processes of fabric and textile back‐injection molding are most commonly used to achieve a bond between the textile and a plastic substrate. With this technique stability and functionality like latching features for assembling in the car are given to the part. When textiles are back‐injected, a material bond is created which is inseparable. This makes recycling much more challenging causing that fabrics and composites that cannot be recycled in the material sense but usually are sent for thermal recycling, no longer available for a circular economy. **Figure** **1** illustrates schematically the back‐injection process and the investigated milling and regranulation (Step 4) to implement a closed loop production.

The majority of fabric‐laminated or back‐injected components utilize PP as a substrate material. Nevertheless, the fabrics employed in these applications are typically composed of polyester, which exhibits immiscibility with polyolefins. This incompatibility leads to an unsuitable material for reuse in automotive interior applications if regranulated, due to the resultant lack of homogeneity and potential degradation of material properties.^[^ ^10^ ^]^ From a recyclability perspective, a viable approach could involve selecting plastics from a polymer family that exhibits enhanced compatibility with polyester‐based fabrics. In principle, this strategy would yield improved recyclability outcomes. A promising class of materials that, in addition to fulfilling the technical requirements related to processing, thermal, and mechanical stability, would be the above‐mentioned technical polymers and their corresponding blends. Therefore these materials are used as a standard for functional and decorative parts in automotive interiors and meet technical and aesthetic requirements. This makes them interesting for future applications as textile covered carrier parts. Over the past few years a number of studies about the recyclability and the properties of these materials have been published. The focus of these studies did lie on the recycling of parts with different coatings, like chrome, or painting.^[^ ^11^ ^]^ Other studies known from literature have focused on combinations of PET and PP, with PET serving as the reinforcing material,^[^ ^12^, ^13^ ^]^ or the recycling of painted PP parts in the exterior.^[^ ^14^ ^]^ However, till now no scientific work examined the recycling of fabric back‐injected plastic panels with PC based carrier material aiming of reusing them in the original pore process.

This work investigated the mechanical recycling of fabric‐back‐injected decorative components with the aim of using the resulting regranulate for reuse as a carrier material for the fabric‐back‐injected decorative components. Specifically, material combinations were investigated that ensure the best possible chemical compatibility and thus a homogeneous mixture of the PET fabric with the plastic carrier material in the recycled granulate. The focus of the investigations was on the thermal, rheological, mechanical, and morphological properties and changes. The objective is to gain a deeper understanding of how the combination of these two materials influences the recyclability and subsequent properties of the regranulated materials.

## 2 Experimental Section

### 2.1 Materials

**Figure 2:**
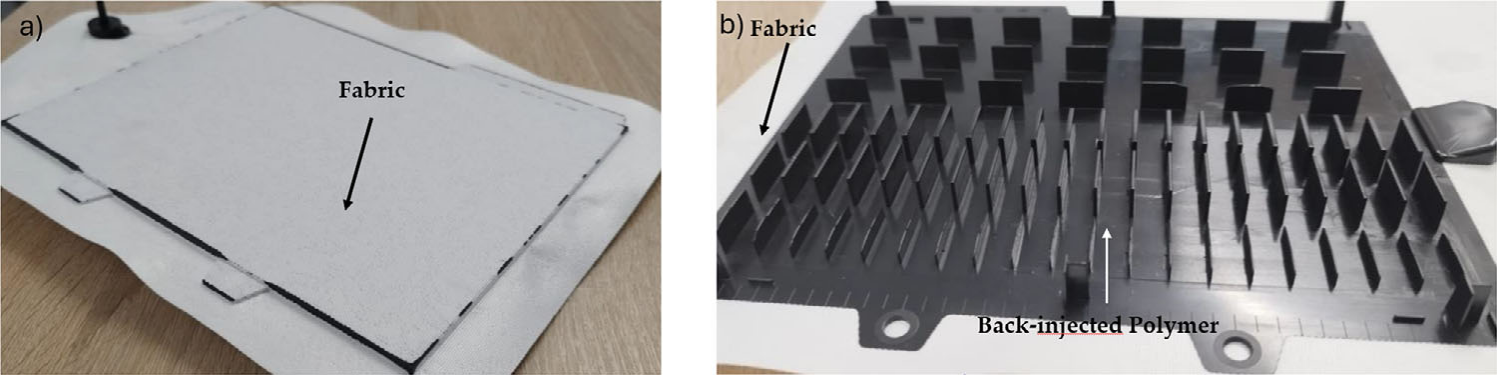
Component used in this work. a) Top view with the fabric, b) back view showing the structure of the back‐injected polymer part.

**Figure** **2** illustrates the structural configuration of a back‐injected component utilized in this study. The sample plate is originally designed to investigate the effects of variations in viscosity and shrinkage of thermoplastic polymers, therefore the rigid component features multiple tears with diverse orientations and thicknesses. For this study it was used to simulate a rigid thermoplastic carrier for a back‐injected textile fabric. The textile coated sample plate was shredded and the ground material was regranulated as described below. The pellets were used to produce testing samples like tensile bars via injection molding. The subsequent discussion will focus on the commonly employed materials for automotive parts based PC, which are used for decorative trim parts.

The fabric was a polyester based on PET provided by the company Carl Weiske GmbH & Co. KG. It had a basis weight of 450 g m^−^^2^.

For the back injection molding process, three commercially available PC based materials were chosen.

Makrolon 2407 from Covestro is a PC, which is utilized in its standard black coloration. It has been specifically designed for injection molding applications, featuring UV stabilization with a deforming agent to enhance its processing properties.

Pocan C1206 from Envalior is a PC/PET blend, which was supplied in standard black coloration.

Bayblend T85XF (BBT85XF, PC/ABS), a high‐performance material from Covestro, is distinguished by its good impact strength and hardness.

### 2.2 Methods

#### 2.2.1 Back‐Injection Process

The production of fabric back‐injected trims was carried out on a ClassiX CX 160–750 injection molding machine from KraussMaffei, which features a 3‐zone screw with a diameter of 40 mm and a maximum clamping force of 1600 kN. The material‐specific processing temperatures recommended by the manufacturer were used for each material. Processing temperatures were kept at 260–275 °C for BBT85XF, 255–280 °C for Pocan C1206, and 275–300 °C for Makrolon 2407. The mold temperature was kept at 80 °C.

#### 2.2.2 Milling and Regranulation

The injection‐molded parts were weighed with a scale with and without fabric to determine the amount of PET in the textile fabric coating. The weights were found to be ≈134 g for the bare parts and approx. 157 g for the parts with fabric. This corresponds to a fabric weight of approx. 23 g per component, which results in a PET content of approx. 15% in the final blends. After injection molding of the sample plates, the test specimens were cut to size and then shredded. This resulted in particles with varying sizes of between 4 and 15 mm.

**Figure 3:**
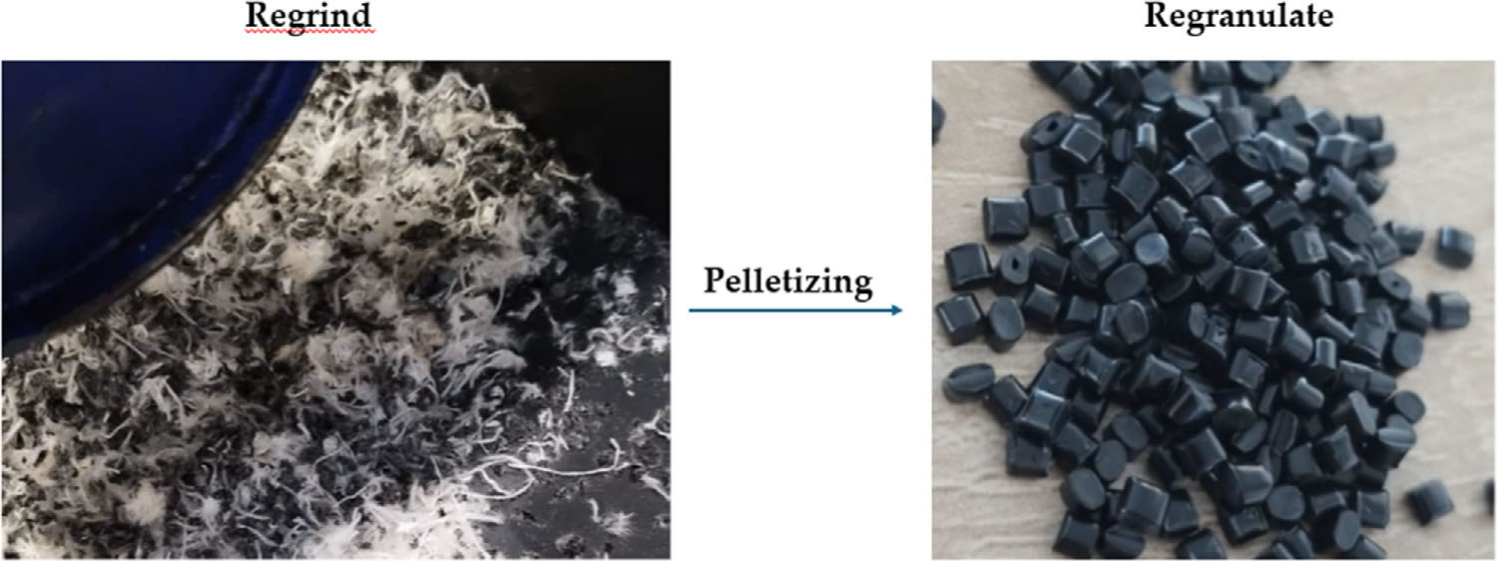
Image of the regrinded parts and the final pellets.

The TG20 thermal pelletizing system from Wanner Technik GmbH was used to transform the shredded test specimens into a processable granulate. The system was equipped with a single‐screw for extrusion, with an additional small counter‐rotating screw in the feed zone, to enhance the feeding of the regrind material. The L/D ratio of the main single screw was 16. The extrusion speed was set to 18%–20% of max. power, the dye pressure was between 15% and 20% of max pressure. Specific values were not able to set since the machine works with percentages. The molten material was then cooled in a water bath at a constant temperature of 11 °C and subsequently granulated. The milled parts and regranulated pellets can be seen in **Figure** **3**. The regrind was dried before regranulation to prevent moisture‐related problems. The drying conditions for materials with fabric content were 4 to 5 h at 100°C for BBT85XF+PET, 7 to 8 h at 100°C for Pocan+PET, and 7 to 8 h at 120 °C for Makrolon+PET. The regranulation and compounding process temperatures (feeder to dye) were 260–280 °C for Makrolon+PET, 250–265 °C for Pocan+PET and 250–265 °C for BBT85XF+PET. Extrusion speed of the single screw was set 20% of maximum power.

#### 2.2.3 Injection Molding of the Test Specimens

Test tensile bars and rheology test plates were produced by injection molding using neat and regranulated material. To produce tensile bars, an Allrounder 420C injection molding machine from Arburg was used. The rheology test plates, on the other hand, were manufactured using a BOY XS from Dr. Boy GmbH & Co. KG. All materials were dried at 100–120 °C for 12 h. Injection molding was conducted with barrel temperatures ranging from 255 to 275 °C for Bayblend T85XF (BBT85XF) and BBT85XF+PET, 255 to 280 °C for Pocan C1206 and Pocan+PET, and 255 to 300 °C for Makrolon 2407 and Makrolon+PET. Mold temperatures were maintained at 80 °C for tensile test specimens and 20 °C for rheology plates.

### 2.3 Analytical Methods

#### 2.3.1 Differential Scanning Calorimetry (DSC)

For DSC analysis the DSC 200F3 Maia from Netzsch was used. Samples from the virgin material as well as the regranulates were weighed to 10 ± 1 mg and pressed into crucibles. Heating was carried out at 10 °C 𝑚𝑖𝑛^−^ from 25 to 300 °C in an inert gas atmosphere of N_2_. The nitrogen gas was fed into the furnace chamber at 50 𝑚L 𝑚𝑖𝑛^−1^. The samples were analyzed with two heating curves. The duration of the isothermal phase after the first heating and before the cooling curve was 5 min for samples from the blends. For the samples of the pure textile, the duration of the isotherms was increased to 10 min to ensure complete melting of the fibers.

#### 2.3.2 Transmission Electron Microscopy (TEM)

The morphology of the blends was analyzed using transmission electron microscopy. The samples required for this were taken from an injection‐molded tensile test bars using microtome cutting. The microtome device used for the cutting was a LEICA EM UC7 from company Leica Mikrosysteme GmbH and used a diamond knife. The samples were cut perpendicular to the injection molding direction, from the core area of the tensile bars. This allowed for analysis of the morphology of the materials without taking into account any influences from the injection molding process, such as shear‐induced stretching of blend phases in the edge layers. The thickness of the sections was 50 µm. The thin TEM samples taken in this way were then contrasted with RuO_2_ for 15 min at atmospheric pressure. The transmission electron images were taken using a JEM‐2200FS device from Joel.

#### 2.3.3 Rheological Measurements

Rheological characterization of the regranulated compounds was performed to investigate their viscoelastic properties. All measurements were conducted using an TA Instruments HR‐20 rheometer, equipped with a plate‐plate geometry configuration (diameter: 25 mm). The compressed film discs prepared according to the protocol outlined in Section 2.2.3 served as test specimens. To ensure thermal equilibration, a TA Instruments Smart Swap ETC oven with convection heating was used. Prior to conducting frequency sweep measurements, preliminary tests were performed to assess thermal stability (isothermal time‐dependent measurements) and to determine the linear viscoelastic region through strain sweep tests. The rheological properties of the test specimens were evaluated using strain sweep and frequency sweep tests at temperatures of 250 and 280 °C. Strain sweeps were conducted at a frequency of 1 Hz and an elongation range of 1%–30% to determine the linear viscoelastic regime at each temperature. Frequency sweeps were performed at a constant strain of 1% and a frequency range of 0.1–30 Hz.

#### 2.3.4 Tensile Tests

The mechanical properties of the blends were analyzed using tensile tests. The tests were conducted in accordance with DIN ISO 527 to determine the characteristic mechanical properties of the materials and to compare these with those of the pure material. Tensile testing of the injection‐molded tensile bars was performed using a Z020 universal testing machine from Zwick Roell. The tests were conducted with a test speed of 50 mm min^−1^, a preload of 10 N, and a maximum test load of 20 kN at a constant temperature of 23 °C.

## 3 Results

### 3.1 Transmission Electron Microscopy TEM

#### 3.1.1 Results for Macrolon 2407 and Makrolon+PET Regranulated

**Figure 4:**
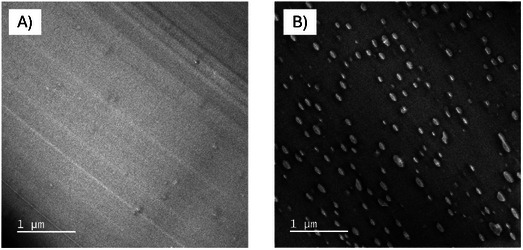
TEM images of Makrolon 2407 A) and Makrolon+PET B); textile PET from droplet phases (light grey) with dimensions < 500 nm.

**Figure** **4** shows the images of the Makrolon 2407 (A) and the Makrolon+PET (B). The PC showed a single‐phase system with a homogeneous morphology (Figure 4A). Compounding with the textile PET, however, lead to the formation of a two‐phase morphology (Figure 4B). Here, the formation of a droplet morphology was observed, with the PET (light phases) being very homogeneously dispersed and distributed in the surrounding PC matrix phase. The phase size of the PET droplets was well below 500 nm if the scale bar was considered, which indicates good compatibility between PC and PET.^[^ ^15^ ^]^

#### 3.1.2 Results for Neat Pocan C1206 and Regranulated Pocan+PET

**Figure 5:**
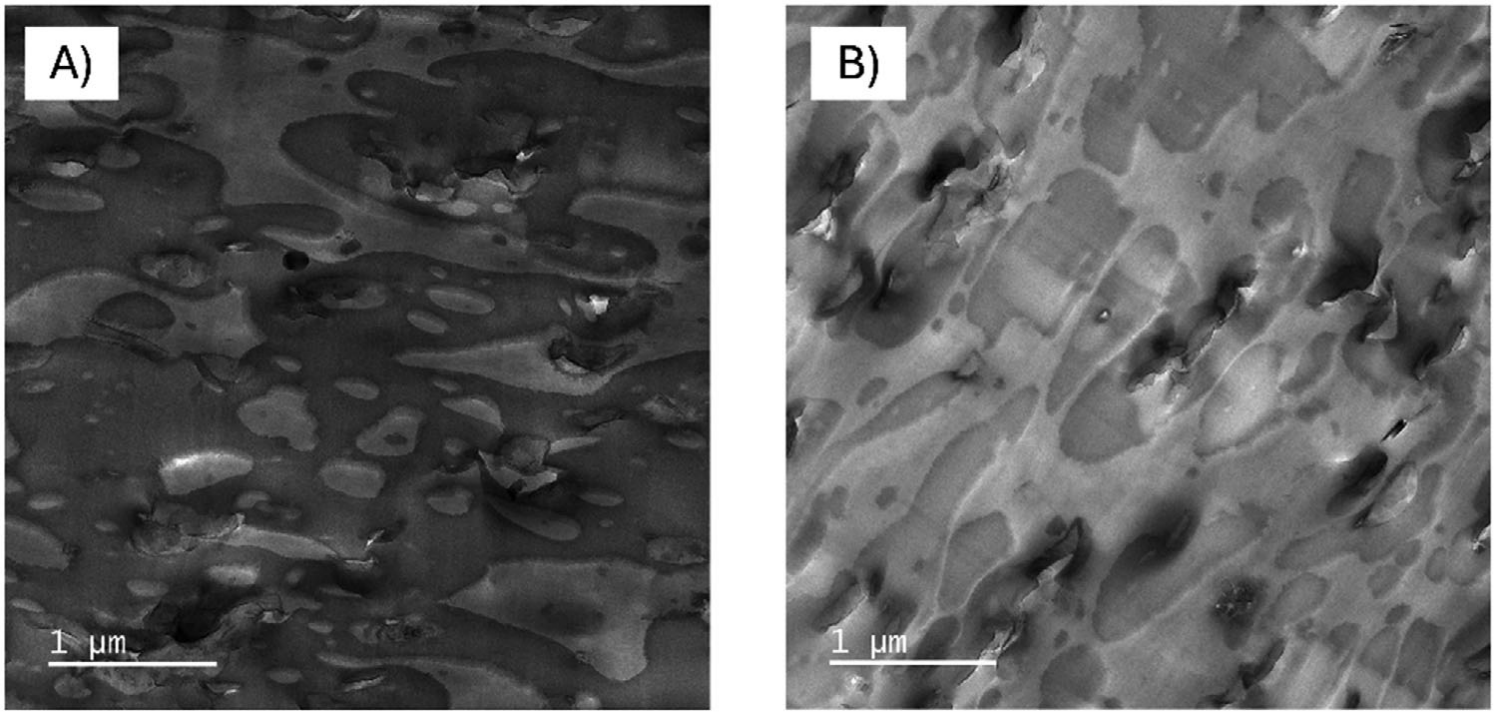
TEM images of Pocan C1206 A) and Pocan+PET B); the addition of textile PET leads to the change.

The TEM analysis of the Pocan C1206 used in this work showed a co‐continuous morphology (see **Figure** **5**A). A co‐continuous morphology exists when the respective phases are present in larger, connected morphologies and penetrate each other, similar to 3D networks. The PC was visible in the images as a dark gray phase, the PET as a light gray phase. When the proportion of PET in the blend was increased by compounding with textile PET, the point of phase inversion of the blend is exceeded and the PET formed a matrix in which the PC is dispersed (see Figure 5B). Wide and long phases of the PC are visible, which represent a mixture of lamellar and coarse droplet morphology within the PET matrix.

#### 3.1.3 Results for Neat BBT85XF and the BBT85XF+PET

**Figure 6:**
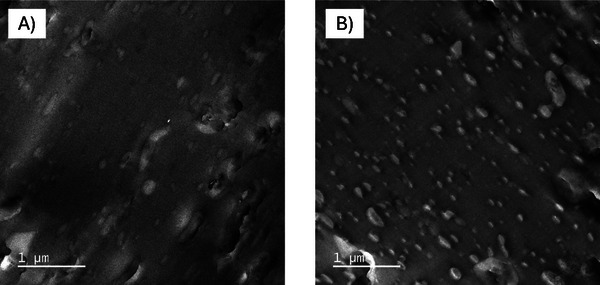
TEM images of BBT85XF A) and BBT85XF+PET B); textile PET from droplet phases (light grey) with dimensions < 500 nm.

**Figure** **6** shows the images of the BBT85XF (A) and BBT85XF+PET (B). The BBT85XF showed the expected morphology of a PC matrix phase (dark gray) and a well dispersed droplet ABS phases (light grey). After the textile PET fabric was introduced into the BBT85XF during the compounding process, the increased number of light gray, dispersed phases suggested that the textile PET is distributed homogeneously in the PC matrix. The phase size of the PET droplets was less than 500 nm if the scale bar was considered, which indicates good compatibility of the PET with the PC. This finding was comparable to the results that were observed with Makrolon 2407.

The contrasting method used in this work couldn't have been used to determine whether a finer dispersion of the ABS phases in the PC matrix also occurs. However, the larger brighter phases visible in the Figure 6B), which are similar to those in pure BBT85XF, suggest that PET has no influence on the dispersion and distribution of the ABS phases.

### 3.2 Differential Scanning Calorimetry (DSC)

DSC analysis provides insight into whether the produced regranulate blends are non‐miscible, partially miscible, or fully miscible. The properties and characteristics of the blend are influenced by this behavior. To determine the pure material characteristics, the second heating curves are utilized in the following analysis. These curves are representative examples of individual measurements. Initially, the PET fabric was analyzed to primarily determine its glass transition temperature (Tg). Subsequently, the second heating curves of the pure granulate was compared with those of the blend and examined with regard to their Tg values and potential shifts. Also, the melting behavior of the crystalline phases of PET was investigated.

#### 3.2.1 Result of the PET‐Fabric

**Figure 7:**
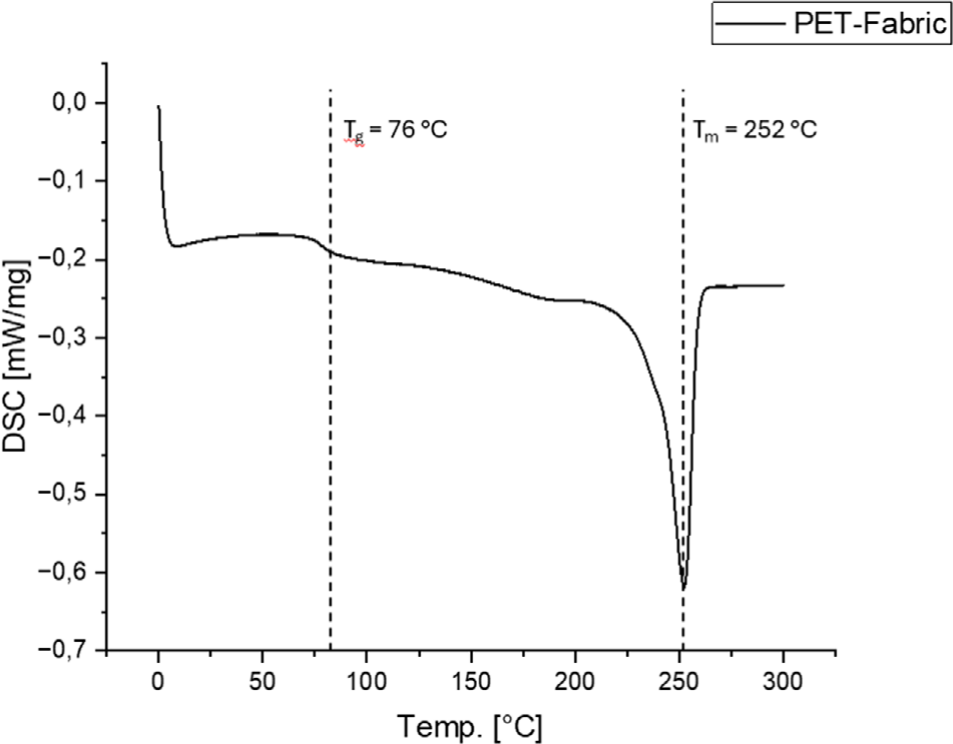
DSC curves of the neat PET‐fabric.

**Figure** **7** shows the second heating curve of the pure PET material. The glass transition temperature Tg of the fabric was 76 °C and the melting temperature Tm was 252 °C. The curve showed no conspicuous features and represented typical behavior for textile PET. This can be found in the work of De Clerck et al,^[^ ^16^ ^]^ which investigated the thermal properties of PET fibers as a function of the process conditions.

#### 3.2.2 Results of Neat Macrolon 2407 (PC) and Regranulated Makrolon 2407 + PET

**Figure 8:**
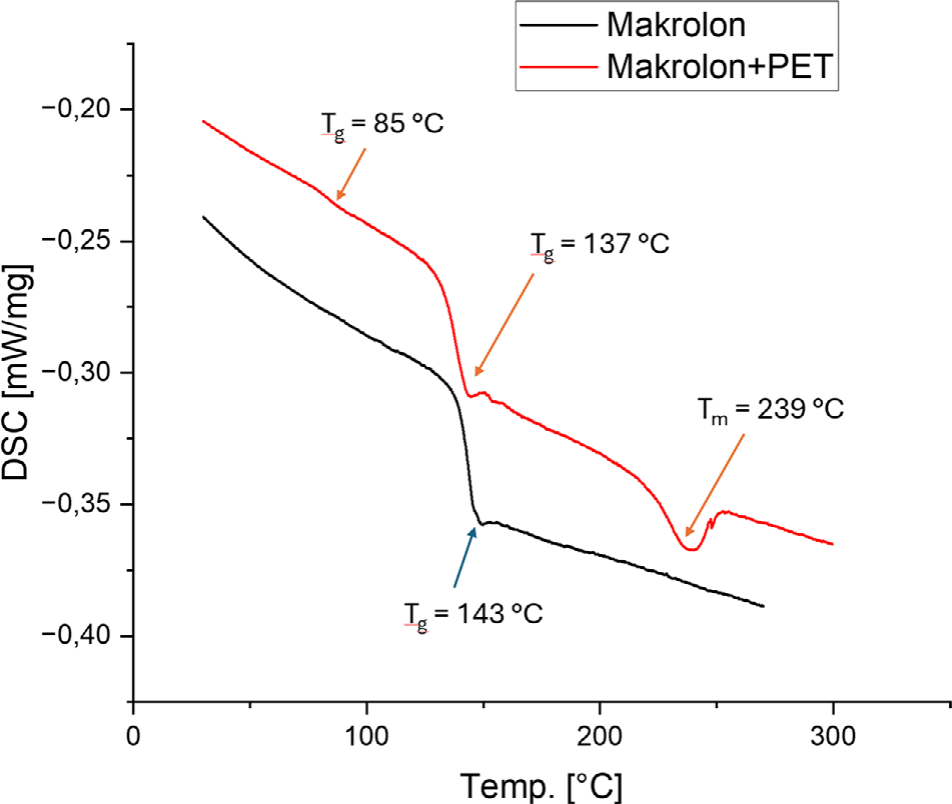
Comparison of the second heating curves between Makrolon 2407 and Makrolon 2407 + PET‐fabric.

According to Cook et. al., the glass transition temperature of polycarbonate is 143 °C and that of PET is 75 °C.^[^ ^13^ ^]^ **Figure** **8** shows that the glass transition temperature of the polycarbonate droped from 143 to 137 °C in the blend with textile PET. The T_g_ of the PET at 85 °C was slightly higher compared to the T_g_ of the neat textile PET fabric. Therefore, a shift of the glass transition temperatures toward the T_g_ of each blend partner was identified on the basis of both of the pure materials. This was interpreted as a tendency towards partial miscibility of the blend produced and was supported by the results of TEM analysis which showed a fine dispersed PET droplet phase in the PC matrix. The melting temperature of the PET phase showed a decrease of approx. 13 °C compared to the neat textile PET. The reason for this behavior might have been due to small droplet sizes of the PET phase and therefore a hindered formation, or a change of crystalline structures. The fact that the peak also showed a broader base supports this theory. This results in a reduced thermal energy requirement for melting the PET, thereby decreasing the melting temperature.

#### 3.2.3 Results of Neat Pocan C1206 (PC+PET) and Regranulated Pocan C1206 + PET

**Figure 9:**
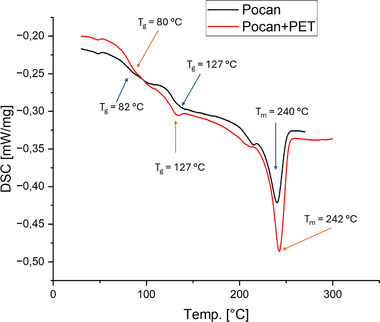
Comparison of the second heating curves between Pocan C1206(PC+PET) and Pocan C1206 + PET fabric.

**Figure** **9** shows a representative second heating curve of Pocan as a pure material and as a blend with the textile PET.

The glass transition temperatures of PET and PC did not change significantly after adding textile PET. However, the T_g_ of PC was lower and the T_g_ of PET was higher compared to the glass transition temperatures of the neat textile PET and the neat Makrolon 2407. This indicates that PC and PET of the Pocan grade showed a partially miscibility in the amorphous phases. This phenomenon was analogous to the effect observed on the T_g_ of PC when textile PET was incorporated, resulting in a shift of the glass transition temperatures.

The melting temperature of the PET phase didn't change, however, the size of the melting peak increased for the Pocan+PET blend. This was due to the additional PET in the blend which increased the total amount of crystalline phases within the blend material.

#### 3.2.4 Results of the Neat BBT85XF and the BBT85XF+PET Regranulate

**Figure 10:**
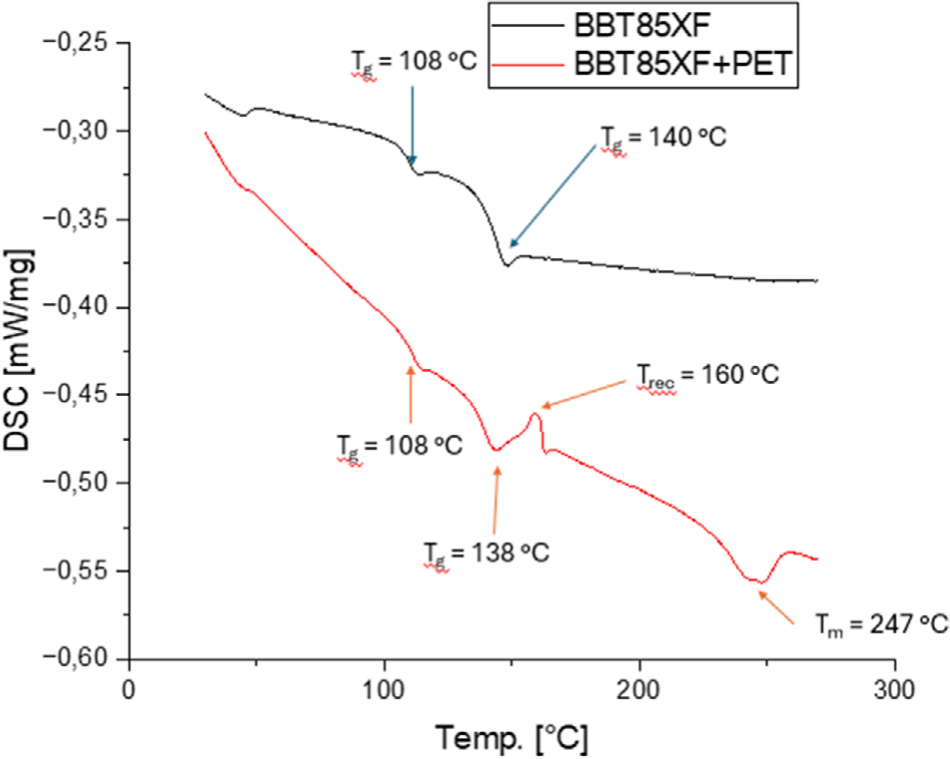
Comparison of the second heating curves between BBT85XF and BBT85XF + PET‐Fabric.

**Figure** **10** shows two representative second heating curves of BBT85XF and the blend with PET material.

The glass transition temperature of PC was found to be 140 °C and the one of the ABS phase at ≈108 °C in the neat BBT85XF. By adding textile PET the T_g_ of the PC phase was slightly reduced to 138 °C, which was comparable to the results found in Makrolon+PET blends. It should also be mentioned that the glass transition temperature of PET, which according to Figure 7 should be 76 °C, could not have been detected. This might have been due to the low content of PET within the blend. However, the exothermic peak in the blend curve at T_rec_ = 160 °C was based on the recrystallization of the PET. Cook et al^[^ ^17^ ^]^ have also demonstrated this recrystallization peak in ABS/PET blends using DSC analyses. It is assumed that this peak is caused by a superposition of the glass transition temperature of SAN from the ABS and the recrystallization of the PET. The semi‐crystalline PET also produces a melting peak at approx. 247 °C, which clearly demonstrates the presence of the PET phase. If the melting temperature of the PET in the textile was compared with that of the PET in the blend, it was noticed that this fell in the blend from approx. 252 °C to the 247 °C, which was a shift of approx. 4–5 °C. Based on the broadness of the peak the reason for the decrease of the melt temperature is the different crystalline structure of the PET.

### 3.3 Rheological Measurements

In the following, the rheological changes of the regranulated materials blended with ≈15% PET fabric were analyzed by frequency sweep measurements in the linear regime. The results of the frequency sweep measurements at 250 and 280 °C are presented, as these temperatures reveal the most significant differences between the neat and regranulated polymer materials.

#### 3.3.1 Results for Macrolon 2407 and Makrolon+PET Regranulated

**Figure 11:**
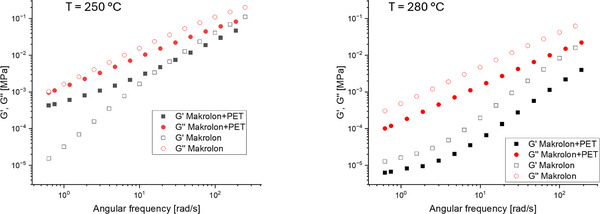
Differences of the storage (G’) and loss (G’’) modulus of the neat and the regranulated Makrolon 2407 measured in the linear regime at 250 °C and 1% strain.

**Figure** **11** illustrates the rheological response of Makrolon 2407 and Makrolon+PET. A distinct difference in rheological behavior is observed between the blended and neat samples. As TEM analysis has shown the textile PET was well dispersed in the PC matrix and according to the DSC measurement had a melting point of 239 °C. Therefore, at 250 °C the dispersed PET was supposed to be almost completely molten considering DSC results, however it did not show an significant influence on the rheological behavior since the viscosity of the PC was dominating. At 280 °C however the molten PET acts as plasticizer and therefore decreases the viscosity of the Makrolon+PET compared to the neat Makrolon 2407.

#### 3.3.2 Results for Pocan C1206 and Regranulated Pocan+PET

**Figure 12:**
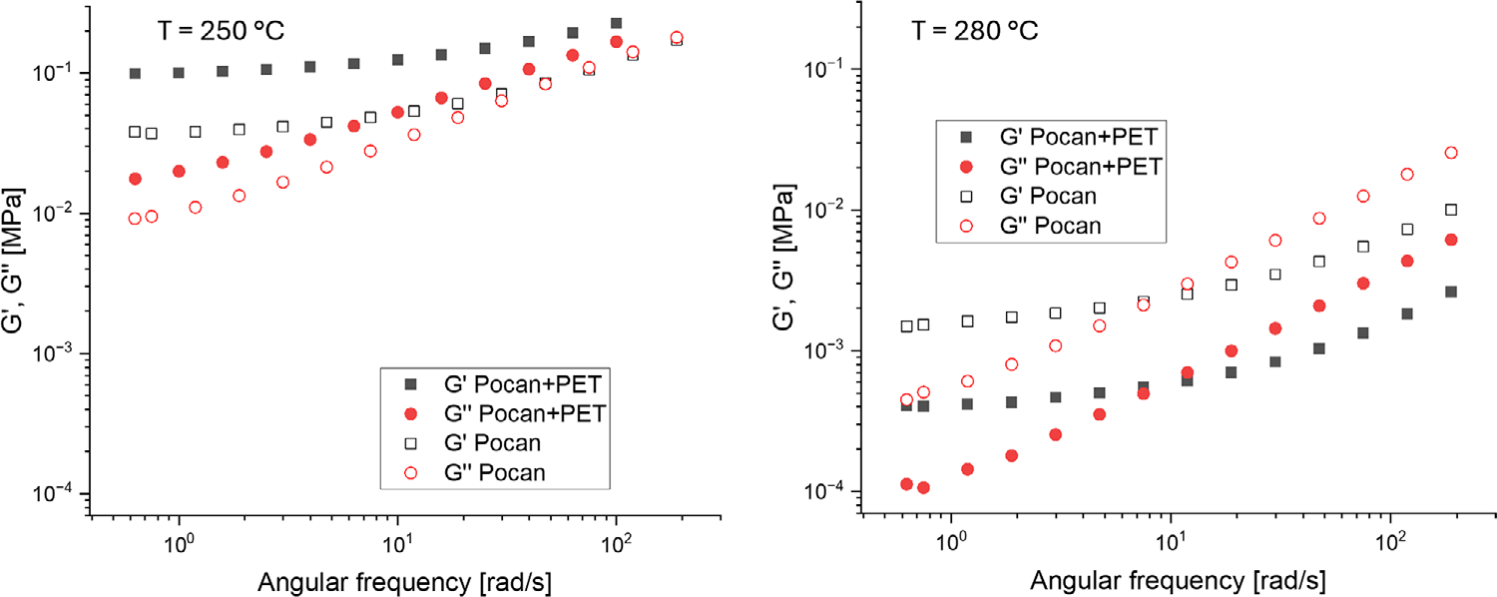
Differences of the storage (G’) and loss (G’’) modulus of the neat and the regranulated Pocan C1206 measured in the linear regime at 250 °C and 1% strain.

**Figure** **12** presents the rheological response of neat and regranulated Pocan C1206 samples. The modulus values of the regranulated sample surpassed those of the pristine sample at 250 °C, with the discrepancy being more pronounced at lower frequency regimes. The cause of such behavior was to be found in the morphological structure of the regranulate material. As can be seen in Figure 5, PET forms a matrix and PC became the dispersed phase in the Pocan+PET blend. With a melting point of 242 °C the PET was not fully molten yet at 250 °C and therefore especially at lower frequencies showed a strong increase in viscosity compared to the neat Pocan C1206 which had a co‐continuous morphology. At 280 °C however the PET was fully molten and therefore the Pocan+PET had a significant lower viscosity compared to pristine Pocan C1206.

#### 3.3.3 Results for Neat BBT85XF and the BBT85XF+PET

**Figure 13:**
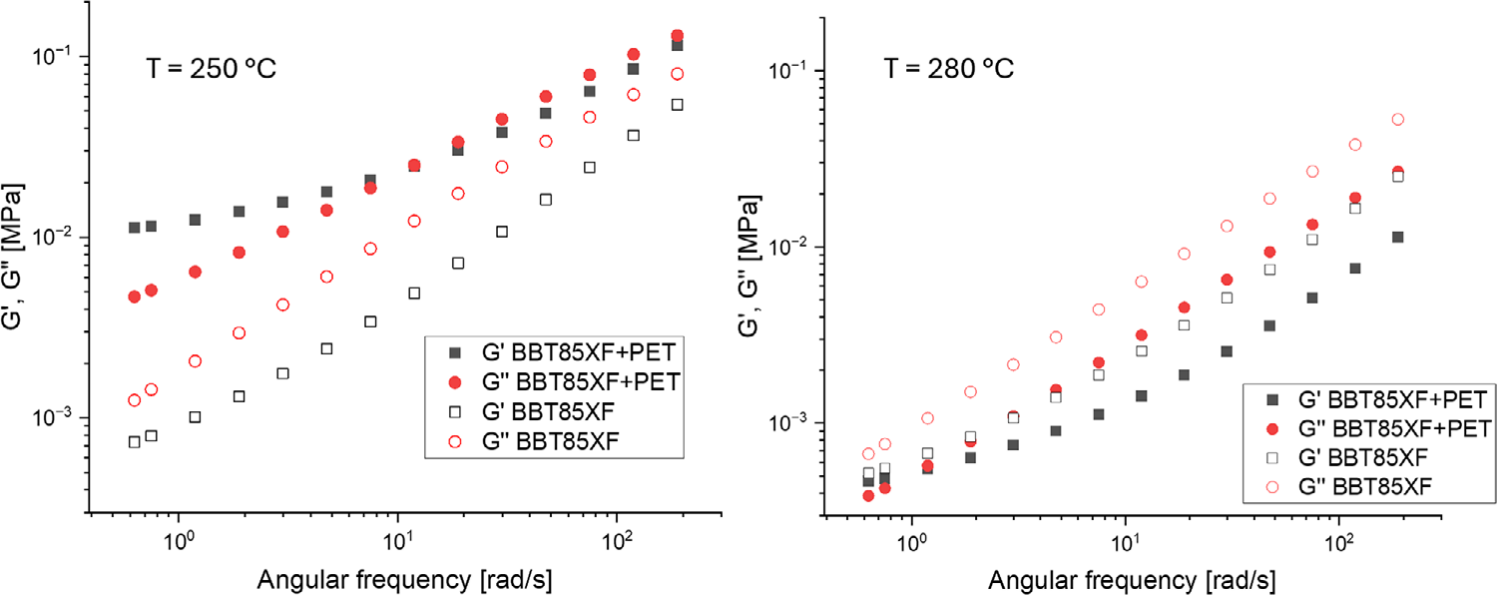
Differences of the storage (G’) and loss (G’’) modulus of the BBT85XF and BBT85XF+PET were measured in the linear regime at 250 and 280 °C at 1% strain.

**Figure** **13** presents the storage modulus (G') and loss modulus (G'') for neat BBT85XF and regranulated BBT85XF blended with PET fabric. At 250 °C both G`and G´´ of BBT85XF+PET lie above of those of the neat BBT85XF, whereas at 280 °C the regranulated material showed a lower viscosity compared to the neat one. This behavior was explained with results from TEM and DSC analysis. TEM analysis showed that the textile PET was dispersed as droplets with diameters less than 500 nm in size in the PC matrix. Also DSC showed that the textile PET fraction in BBT85XF+PET has a melting point of 247 °C. This resulted in the PET droplets behaving as a shear‐thickening additive to the melt at 250 °C, thereby increasing the viscosity. At 280 °C however, PET is completely molten, and due to the low intrinsic viscosity of textile PET it lowers the viscosity of the regranulate materials compared to neat BBT85XF. This effect is comparable to the results of the Makrolon based materials. However, in BBT85XF there was als ABS as second, third phase respectively. The presence of ABS also had an effect on the rheological behavior of both the neat BBT85XF and the blend with textile PET. In general, ABS shows a shear thinning rheological behavior throughout a wide range of shear rates. In this study, this lead to the effect that the influence of textile PET on the flow behavior was not as pronounced as it was the case with the Makrolon+PET blend system.

### 3.4 Mechanical Measurements

**Figure 14:**
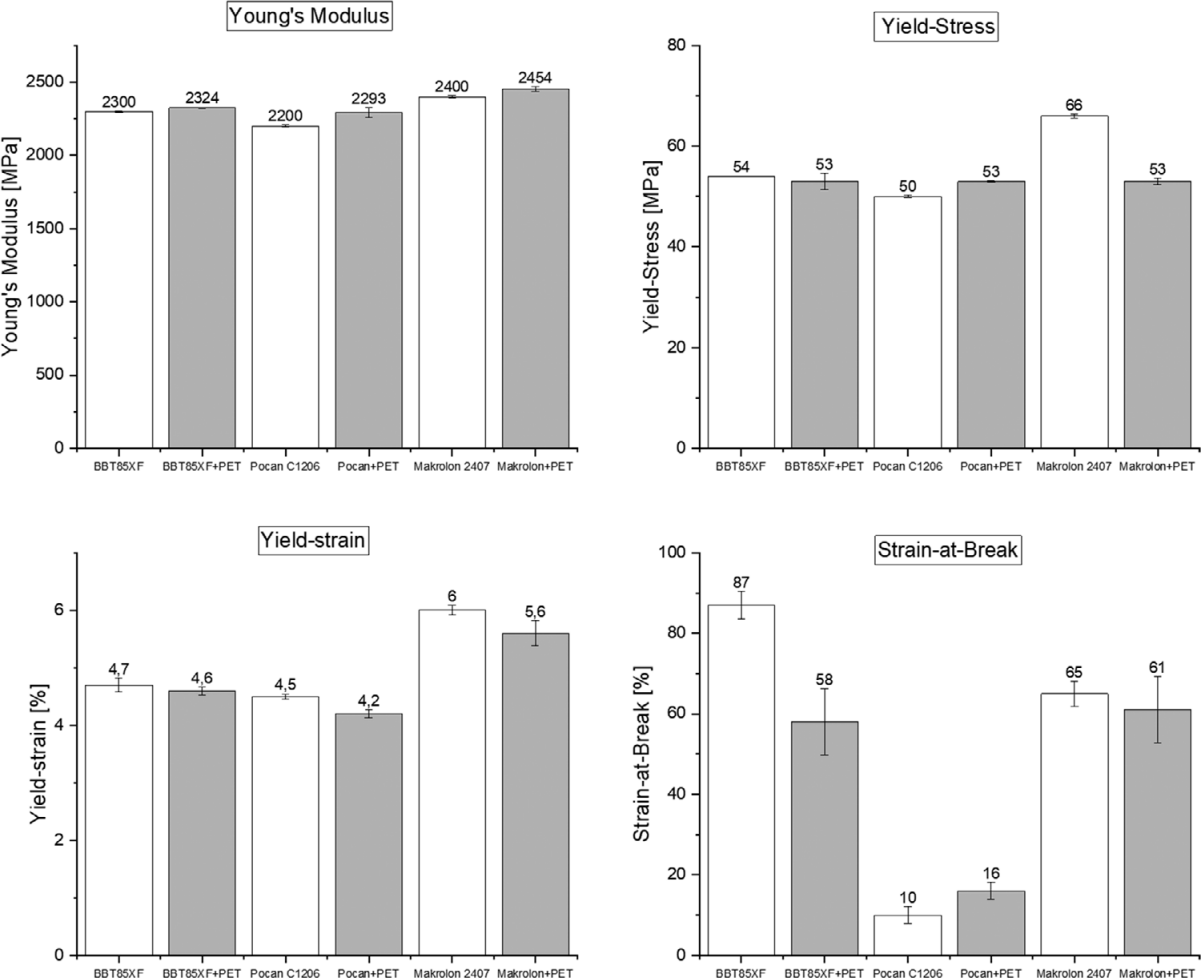
Results of the tensile tests for the neat (hollow bars) and the regranulated (filled bars) polymer samples. To characterize changes in the mechanical properties the E‐Modulus, yield‐stress, yield‐strain, and the stress‐at‐break were used.

**Figure** **14** shows the mechanical properties of the neat and regranulated polymer samples, represented by the Young's Modulus, yield‐stress, stress‐at‐break, and strain‐at‐break.

For the Young's Modulus, which represents the stiffness of the materials, there was no significant influence of the textile PET found, when it was compounded with the three PC‐based materials used in this study.

The yield stress (yield strength) of the three blends with textile PET showed the same values with ≈53 MPa with a very small standard devation. For BBT85XF the yield strenght was almost exactly the same for both the neat material and the textile containing blend. The yield strenght of the Pocan+PET blend was slightly increased compared to the neat Pocan which could have been an effect of the phase inversion of the blend shown in TEM results and the higher crystallinity that was seen in DSC analysis. Only Makrolon+PET suffered form a more distinct decrease in yield strength, when textile PET was compounded with it. This decrease has to be investigated in further studies, but it was assumed that that the fine dispersion of PET in the PC matrix lead to stress concentrations in boundarie layers between PC and PET and therefore to an earlier plastic deformation of the PC matrix which results in a decrease in yield strength.

Though Makrolon+PET showed this decrease in yield strength, yield strain and strain‐at‐break were similar to neat Makrolon. This supports the results from DSC analysis which indicated a partial miscibilty of the materials, because no decrease in strain‐at‐break was detected which would have occurred due to a critical stress concentration in the interphase between PC and textile PET. A slight increase in strain‐at‐break was observed for Pocan+PET which might have been due to the same effect as the increase in yield strength was found. The additional textile PET lead to a dominating PET matrix, as it was shown in TEM results. This lead to the effect of an improved plastic deformation of the material and therefore a higher strain at break. For BBT85XF however, a distinct decrease of strain‐at‐break was observed, when textile PET was compounded with it. The reason for this has to be investigated in future studies. For this study, it was assumed that the small textile PET droplets within the PC matrix hindered the ABS phase from plastic deformation at higher strain rates. This might have caused a critical stress concentration and let to an earlier critical failure of the material.

## 4 Conclusion

In this study, we explored the structure‐property relationships of polycarbonate (PC)‐based back‐injected polymers combined with polyester textile, focusing on the impact of blending PET fabric with Makrolon 2407, Bayblend T85XF, and Pocan C1206 via single‐screw compounding during the recycling process. TEM analysis revealed that PET formed a droplet morphology (<500 nm domains) in Makrolon 2407 and Bayblend T85XF, while blending with Pocan C1206 induced a phase inversion from co‐continuous to a PET matrix with large dispersed PC phases. DSC results indicated partial miscibility of PET with Makrolon 2407 and Bayblend T85XF, evidenced by shifted glass transition temperatures, alongside increased crystallinity in Pocan. Rheological analysis showed that PET reduced melt viscosity at 280 °C across all three PC‐based materials, enhancing their suitability for injection molding. Mechanically, PET slightly lowered yield strength in Makrolon and strain‐at‐break in Bayblend, likely due to PET droplets promoting earlier plastic deformation and critical stresses, whereas Pocan exhibited modest improvements in mechanical properties, attributed to the PET matrix and enhanced crystallinity.

This work demonstrates that mechanical recycling influences the melting and rheological behavior of PC‐based materials used in automotive interiors, a factor that must be considered in future part design and development. However, limitations in this study suggest the need for further research to fully elucidate the effects of recycled PET blending across diverse polymers and conditions.

## Conflict of Interest

The authors declare no conflict of interest.

## Data Availability

The data that support the findings of this study are available on request from the corresponding author. The data are not publicly available due to privacy or ethical restrictions.
